# A Cross-Temporal Meta-Analysis of Changes in Tourists’ Well-Being in China 2011–2022

**DOI:** 10.3390/bs15030264

**Published:** 2025-02-24

**Authors:** Wei Zheng, Zhaoxiang Ba, Chunfeng Long

**Affiliations:** 1 Department of Tourism, Fudan University, Shanghai 200433, China; 22110500004@m.fudan.edu.cn (W.Z.); tunxi@21cn.com (Z.B.); 2 School of Business Administration, Chongqing Technology and Business University, Chongqing 400067, China

**Keywords:** tourists’ well-being, social change, General Well-Being Scale, cross-temporal meta-analysis

## Abstract

Analyzing the overall trend of changes in the well-being of Chinese tourists and its relationship with societal transformations is essential for understanding the psychological shifts of these tourists. This study utilizes cross-temporal meta-analysis and a time-lagged approach to examine 56 studies employing the General Well-Being Scale as the principal tool for evaluating the well-being of Chinese tourists from 2011 to 2022, with the objective of ascertaining whether trends in well-being levels among Chinese tourists and their macro-social indicators clarify discrepancies in tourists’ well-being. The results demonstrate a strong positive link between the well-being of Chinese tourists and the year, signifying an annual increase. The well-being of Chinese tourists is markedly positively associated with economic conditions (per capita income and consumption levels) and social connectedness (urbanization rate, employment rate, and life expectancy); these five social indicators are essential determinants of its variations. This study contributes by elucidating the trend of well-being among Chinese tourists at the group level and confirming that five categories of social variables significantly influence their well-being. It can aid destinations in enhancing relevant social and economic policies, inventing tourism products, and accelerating the development of the tourism industry, thereby substantially boosting the well-being of Chinese tourists.

## 1. Introduction

China, being one of the world’s main sources of outbound tourists, saw a steady increase in the number of people departing before COVID-19. The country’s spending on outbound tourism led the world for seven consecutive years (from 2013 to 2019) (Gao et al., 2022). Specifically, in 2019, Chinese tourists spent 255 billion dollars on cross-border travel, with the number of outbound trips reaching 6.01 billion. China significantly influences the advancement of the global tourist sector. Well-being is a paramount non-economic result of tourism (Gretzel & Stankov, 2021). In recent decades, tourism research has increasingly concentrated on well-being (Smith & Diekmann, 2017). An expanding corpus of literature indicates that tourism and leisure activities substantially enhance quality of life (McCabe & Johnson, 2013). Western nations are educated, industrialized, prosperous, and highly developed democratic societies, typically distinguished by a focus on individual accomplishment, self-esteem, and personal liberty. China significantly contrasts with the West regarding cultural attributes (Hofstede, 2016). In 2022, the report from the 20th National Congress of the Communist Party of China (CPC) emphasized that the enhancement of citizens’ livelihoods and the improvement of their quality of life are imperative. In a special economic, cultural, and political context, it has given rise to the topic of research on the well-being of Chinese tourists. This topic is crucial for the global tourism market to understand the needs and preferences of Chinese tourists. For cross-border tourism businesses, comprehending and meeting the demands of these tourists is essential to enhance their satisfaction and loyalty.

Tourism and leisure activities are widely regarded as a primary means to enhance individual well-being (L. Su et al., 2021). The expansion of the tourism sector and personal participation in related travel endeavors can enhance the subjective well-being of tourists (Kwon & Lee, 2020). It is widely accepted that participation in travel and leisure activities can alleviate loneliness and facilitate the reconstruction of social relationships, hence improving life expectancy and overall quality of life (Kan et al., 2023). Travel and leisure activities are commonly acknowledged as a method for achieving subjective well-being. Through engaging in and experiencing travel and leisure activities, one can alleviate stress, boost physical activity, enhance social connections, broaden their knowledge, and ultimately achieve a heightened sense of personal well-being (Samus et al., 2022). Therefore, participating in tourism activities is of great significance for the generation of well-being, and people can achieve the pursuit of well-being through this leisure activity of tourism.

In recent decades, the growth of holiday management and increased disposable money have led to exponential expansion in China’s tourist business. Although people recognize the role of tourism in generating well-being and acknowledge the significance of tourists’ well-being in pursuing a better life (Bimonte & Faralla, 2015), certain aspects of tourism in China might negatively impact the experience of tourists. For instance, the majority of Chinese tourists work within a constrained travel budget, and in Chinese culture, being economical is highly regarded. The absence of a complete annual leave system results in the majority of Chinese tourists traveling during public holidays, such as the National Day Golden Week. Overcrowded tourist sites and insufficient public amenities have compromised tourists’ experiences, satisfaction, and overall well-being (Liang et al., 2021). The distinctive characteristics of the Chinese setting have somewhat eclipsed the impact of tourism on individuals’ subjective well-being. Although several studies have explored the connection between tourism and tourists’ subjective well-being, less research has focused on the specific well-being of Chinese tourists (Zheng et al., 2022). The psychological condition of specific social groups, such as the well-being of the Chinese, is affected by macro-social changes, including economic conditions (S. F. Xin et al., 2020). It was claimed that the impact of significant social transformations on human development should be assessed through two constructs: economic conditions and social connectedness (Z. Xin et al., 2010). Social connectedness refers to an aspect of the individual self that reflects a subjective sense of closeness and togetherness with one’s social environment. Many studies have found an important role for social relationship variables (e.g., social connectedness, social acceptance, social support) in relation to subjective well-being (SWB) (Yoon, 2008). Xu found that economic conditions, urbanization rates, employment, and health substantially affect tourists’ subjective well-being (Y. Xu & Xia, 2014). In fact, urbanization rates, work, and health are important indicators of social connectedness. Therefore, has the tourist-related well-being of the Chinese changed in this context? What are the trends in these changes? What are the underlying social factors?

In order to clarify the changes in well-being among Chinese tourists and the macro-level social factors influencing this well-being, this study selected 56 literature sources published between 2011 and 2022, involving 42,312 tourists. The well-being of Chinese tourists was examined by cross-temporal meta-analysis (CTMA), while macro-social elements affecting their well-being were investigated using time-lag analysis (Twenge, 1997). Through this method, the limitations of traditional meta-analysis in studying time effects are addressed, trends in Chinese tourists’ well-being over time can be revealed, and the relationship between Chinese tourists’ well-being and social indicators can be analyzed to reveal how social change affects group psychological development. Twenge (Twenge, 2000; Twenge et al., 2016; Twenge & Campbell, 2001) utilized this methodology to examine many psychological factors across time, including anxiety, self-esteem, and well-being. Currently, only a few scholars have used this method to study the well-being changes of specific groups such as teachers, university students, and urban residents, and there is no cross-temporal meta-analysis of the overall well-being changes of Chinese tourists and the social changes that affect tourist well-being under the background of economic development and social reform. Macro-social factors are determined by the distribution of money, power, and resources at global, national, and local levels (S. Zhang et al., 2023). This paper will examine the macro-social determinants influencing tourist well-being by cross-temporal meta-analysis.

## 2. Literature Review

### 2.1. Research Status of Tourists’ Well-Being

Good psychological functioning and experience are the multi-faceted components that make up well-being (Ryan & Deci, 2001).The notion of well-being has historically developed from two philosophical schools: hedonism and eudaimonism (Waterman, 1993). The former highlights the enjoyment derived from certain activities, whereas the latter pertains to the significance of life (Ryan & Deci, 2001). Pleasure is a more direct emotional experience, while happiness is focused on the attainment of meaning and purpose (Vittersø, 2016). Subjective well-being (SWB) encompasses the cognitive and affective assessment of individuals’ lives (Uysal & Sirgy, 2019), incorporating emotional responses to occurrences and cognitive evaluations of contentment and accomplishment (Snyder & Lopez, 2009). Subjective well-being (SWB) highlights the personal aspects of well-being, including feelings and moods (Kesebir & Diener, 2008). It encompasses both positive and negative consequences, in addition to general and domain-specific life satisfaction. Positive effects denote favorable emotions and sentiments, including joy, curiosity, engagement, and affection, which signify an individual’s affirmative reaction to circumstances that suggest an optimal life experience. Conversely, negative effects pertain to adverse feelings and moods, including melancholy, stress, and depression, along with unfavorable responses to life, health, events, and the environment.

In the context of tourism, tourist well-being is a state of positive emotional experiences (joy, interest, satisfaction, and love) and a sense of meaning obtained from engaging in holiday leisure activities (Filep & Deery, 2010). Tourist well-being is regarded as a good sentiment experienced by individuals throughout the tourism journey, encompassing emotional, physical, intellectual, and spiritual dimensions, along with the consequent cognitive awareness (Tuo, 2015). The majority of the expanding corpus of research on tourism-related well-being has focused on subjective well-being (SWB), confirming that tourism has a positive effect on SWB (Mitas & Kroesen, 2020). An exemplary travel experience not only allows tourists to feel relaxed and delighted after the journey but also continuously influences their subjective well-being (L. Xu & Zhang, 2021). The Hedonic Adaptation Theory posits that travel is vital for enhancing a tourist’s subjective well-being, with savoring serving as the primary psychological mechanism in this process (Z. Y. Li & Yan, 2021). This study argues that the concept of tourist well-being combines the core of subjective well-being from both the achievement and pleasure perspectives, emphasizing the positive emotional experience that tourism activities can bring to individuals and their spiritual gains. Although the two research orientations have different emphases, both point to the positive significance of emotions for individuals. This study attempts to combine tourism and subjective well-being to further explain the degree of change in the development of tourists’ well-being.

The study of Chinese tourists’ well-being began in the 21st century (L. Su et al., 2021). After decades of rapid economic development, Chinese people started to pay attention to the issue of well-being, and intuitively feel that tourism can bring well-being to people (Pan, 2014). By evaluating the domestic tourists’ well-being index using Guilin tourists as an example, it was found that the mean (M) score of tourists’ well-being was 3.84, indicating that tourists’ overall sense of well-being was generally moderate (T. Zhang, 2014). With the increase in per capita monthly income, tourists’ life satisfaction has also shown a certain degree of an upward trend (Huang, 2015). With the popularization of tourism, tourists are increasingly pursuing higher quality in their travel experiences, and the level of well-being among tourists has remained stable (Zhan, 2017). Under the policy background of “beautiful countryside”, “targeted poverty alleviation”, and “rural revitalization strategy”, rural areas have become important destinations for mass tourism, and rural vacation tourists have a higher level of well-being (Meng, 2019). Most scholars have discussed the level of tourists’ well-being within a specific time period or with a specific case, and there is limited research regarding the overall trend of tourists’ well-being in recent decades. Therefore, this paper will use the method of cross-temporal meta-analysis to explore the development trend of Chinese tourists’ sense of well-being over the years.

Existing research mainly focuses on the factors that affect tourists’ well-being (L. Su et al., 2019). Studies indicate that for Chinese tourists, their perception of service quality significantly influences their subjective well-being, particularly through their emotional responses and overall satisfaction with a destination. Notably, there is a discernible relationship between subjective well-being and factors like perception, emotion, and satisfaction (He et al., 2020). By examining the “agritourism” model, this study bridges various touchpoints, including tourism experiences, hedonic well-being, intentions to revisit, and online word-of-mouth, offering suggestions on how to respond to the revitalization of rural tourism and attain sustainable tourism development (C. Chen et al., 2021). Based on data from 742 Chinese tourists, within the context of duty-free shopping during their trips to South Korea, it was discovered that the pragmatic, hedonic, and social experiences associated with the products positively affected the tourists’ well-being (Kim et al., 2019a). This study investigates the correlation between intergenerational conflict and parents’ subjective well-being, mediated sequentially by family intimacy and self-efficacy in connection to trip satisfaction (Hu et al., 2023). The above studies explore the factors that affect tourists’ well-being from different micro perspectives, which have certain academic value for different types of tourists and case studies. Nevertheless, limited research exists about the influence of macro-societal changes on the well-being of tourists.

This study integrates the notion of tourist well-being with the correlation between tourism and subjective well-being; however, there is a paucity of research regarding the comprehensive examination of Chinese tourists’ well-being and the underlying factors contributing to its fluctuations. Consequently, the theme of this study is articulated as an investigation into the trends in Chinese tourists’ well-being and the factors that influence these changes.

### 2.2. Macro-Social Factors Influencing Chinese Tourists’ Well-Being

In previous studies, the influencing factors on tourist well-being have mainly focused on demographic variables (Y. Chen et al., 2016) and the changing characteristics of well-being among various types of tourists in China. However, a substantial body of research has demonstrated that tourist well-being, as a psychological variable, is inherently related to social changes (L. Su et al., 2021). China has seen swift progress throughout more than 40 years of reform and opening, attaining substantial achievements in the socioeconomic domain. Economic conditions can affect variations in personal income and consumption levels (Stanca, 2010). The process of industrialization and urbanization has not only absorbed a large number of people into cities, but has also brought new forms of leisure (Clark & Oswald, 1996). In terms of employment, unemployment significantly reduces well-being, and the losses from unemployment go beyond income, with more far-reaching effects on mental health (Z. Xin et al., 2010). With regard to the impact of health on subjective well-being, there is almost unanimous agreement that people in poor health are less happy.

With the increase in urbanization level, employed population, and life expectancy, people’s lifestyles and leisure activities have continuously changed. Tourism, as a form of experiential economy, not only promotes economic growth (Cárdenas-García et al., 2015), but also socioeconomic development encourages more people to participate in tourism to enrich their lives, enhance their experiences, and increase their well-being (Uriely, 2005). Current research has examined the impact of socioeconomic determinants on the well-being of Chinese tourists, focusing mostly on economic conditions, urbanization rates, the employed population, and life expectancy. However, there has been no research conducted on integrating all these factors for comprehensive analysis.

Income functions as a primary catalyst of economic behavior. In economics, it is posited that increased personal income correlates with enhanced happiness. Easterlin shown that individuals in the United States with elevated earnings are more inclined to experience happiness or a high degree of happiness compared to those with diminished incomes (Abramovitz et al., 1974). However, since the “happiness paradox” first emerged, people have begun to realize that having more money does not necessarily make people happier. As income attains a specific threshold, the expenses associated with time and work surpass the revenue generated, resulting in a decline in happiness, hence establishing the concept of “happiness economics.” Although the wealthy in a country may have a higher sense of happiness than the poor, in the long run, happiness does not increase with an increase in a country’s income (Easterlin, 2003).

Income is a primary determinant of consumption patterns. Consumption can promote the generation of individual well-being (X. Wang & Sun, 2019) and good consumption behavior can help enhance well-being (Wu & Yuan, 2012). Cultural and tourism-related consumption projects show a gradually increasing marginal contribution to well-being (Zhou, 2015). Tourism consumption experiences can to a certain extent affect the emotions, sense of participation, and self-realization of tourists (Xie, 2005), thereby increasing their sense of well-being.

Urbanization is frequently affected by income and consumption patterns. As urbanization advances and ecological environment quality is enhanced, the well-being of urban dwellers will rise (H. Li et al., 2019). Simultaneously, urbanization has enhanced individuals’ perceptions of distributive fairness and procedural justice, thereby elevating overall well-being (Jiang et al., 2017).

Employment is closely linked to urbanization and income trends. Employment is regarded as a significant contributor to well-being, since remunerated work is deemed essential for individual welfare by providing cash and satisfying many psychological demands (Stam et al., 2016). Employed individuals typically report more life satisfaction than the jobless, and those engaged in work exhibit markedly higher emotional well-being compared to their unemployed counterparts (Knabe et al., 2017). Moreover, individuals who participate in employment often gain a greater sense of self-esteem and enviable social status (Connolly & Garling, 2022). Increased job satisfaction correlates with a heightened sense of personal well-being (Y. Xu & Wang, 2014).

Health is affected by and influences income, consumption, urbanization, and employment. Health profoundly affects the quality of life and is a vital predictor of personal well-being and happiness (F. Li et al., 2017). Individual health is a crucial contributor to overall well-being, with existing studies indicating that both psychological and physical health positively influence individuals’ well-being (X. Zhang et al., 2017).

The existing literature has predominantly analyzed these elements separately, neglecting to synthesize them into a unified framework that demonstrates their interconnections and overall influence on tourists’ well-being. This study aims to address this gap by conducting a comprehensive analysis of how five macro-social factors—income, consumption, urbanization, employment, and health—interact to affect the well-being of Chinese tourists.

## 3. Methods

### 3.1. Criteria for Scale Selection

Over the past 20 years, nearly 20 measurement tools have been used in studies of tourists’ well-being (W. Zhang et al., 2024). The many scales used to measure tourist’s well-being can be broadly categorized as follows: The initial group often associates Subjective Well-Being (SWB) with happiness (Diener, 2000). The General Well-Being (GWB) Scale, established by (Fazio, 1977), serves as a standardized measurement instrument created for the National Center for Health Statistics in the United States to assess participants’ self-reported well-being. The scale assesses an individual’s experience and equilibrium of happy and negative emotions, alongside their perception and evaluation of health, thereby synthesizing their subjective assessment of quality of life. The second group of scales measures Psychological Well-Being (PWB), developed by Ryff and Keyes (Ryff & Keyes, 1995). The third category of scales is the Multiple Happiness Questionnaire (MHQ), an important theoretical basis for the development of this questionnaire is the unification of SWB and PWB. In summary, the General Well-Being Scale (GWB) is a specialized and comprehensive tool for assessing subjective well-being, characterized by high reliability, frequent utilization, and extensive existing literature employing this scale. Consequently, this study will analyze literature on subjective well-being utilizing the General Well-Being Scale (GWB) revised by Duan, with the mean of the total scores serving as the metric for assessing the level of subjective well-being. A higher score correlates with an elevated level of subjective well-being.

Duan (1996) adapted the GWB scale into a Chinese version, using the first 18 items of the scale to measure. The revised scale includes six dimensions: 1. satisfaction and interest in life (items 6, 11); 2. worry about health (items 10, 15); 3. energy (items 1, 9, 14, 17); 4. mood of depression or happiness (items 4, 12, 18); 5. control over emotions and behavior (items 3, 7, 13); 6. relaxation and tension (items 2, 5, 8, 16). Participants’ statements about happiness are used as the evaluation criterion. Items 1–14 use a 5-point or 6-point scale, while items 15–16 use a 10-point scale. Among them, items 1, 3, 6, 7, 9, 11, 13, 15, and 16 are negative questions, and the total score is obtained by adding the scores of all items after reversing the negative scores. Participants are evaluated based on their emotions over the preceding month, and the mean score of the overall scale is utilized to assess the tourist’s level of well-being. A superior score signifies an elevated level of well-being. Therefore, this article collected original research data on Chinese tourists’ GWB scores for cross-temporal meta-analysis. In order to facilitate the harmonization of measurements and avoid errors caused by different standards, if the scale uses a 6-point or 10-point scale, it is transformed into a 5-point scale according to certain rules (Dawes, 2008).

### 3.2. Literature Search

This article mainly studies the development of and changes in well-being among Chinese tourists. Therefore, the search scope mainly includes three literature databases in China: CNKI, Wan Fang Data, and VIP Chinese Journal Platform. Simultaneously, to guarantee the amount and quality of data, databases such as Web of Science, Wiley Online Library, Scopus, and Taylor & Francis Online are utilized to search for research articles regarding the well-being of Chinese tourists. The search time range is set from 1 January 2000 to 31 December 2022, including journal articles, theses and dissertations, and conference papers. The search was conducted by using keywords such as “tourist well-being”, “tourist happiness”, “subjective well-being”, “China”, and “Chinese” as the main subjects, keywords, and full-text search terms for multiple searches and screenings. In each article, the author, publication year, type of publication, survey area, sample size (N) (including the number of male and female groups), as well as the mean (M) and standard deviation (SD) of the scale, are extracted for cross-temporal meta-analysis.

### 3.3. Inclusive Rules

**Figure 1:**
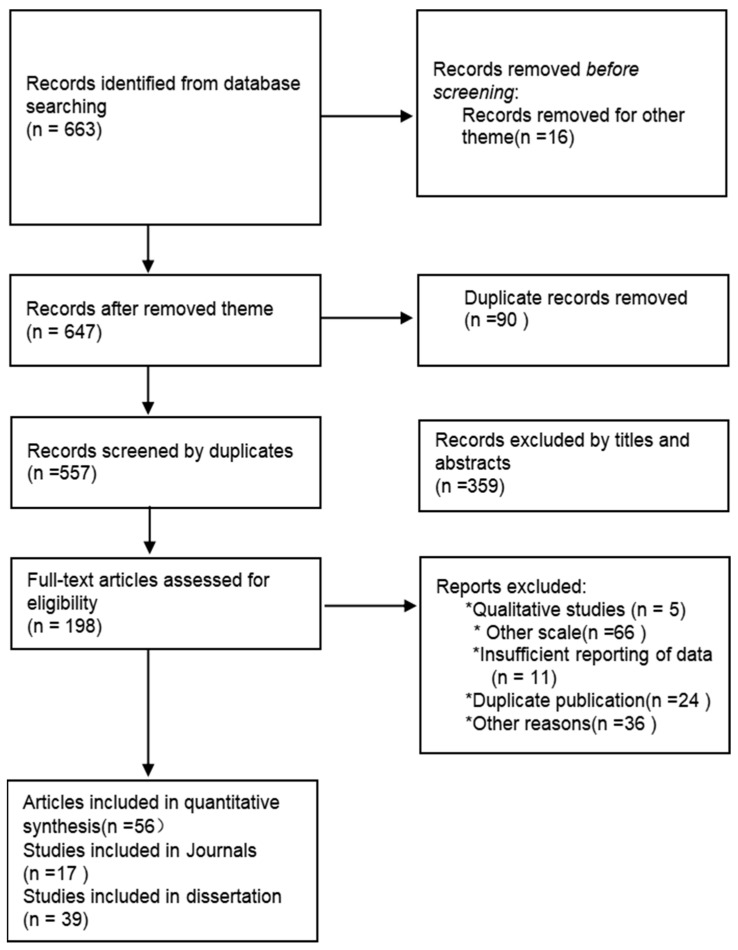
PRISMA diagram illustrating the search strategies and inclusion procedure. Note: * means manual exclusion.

According to Twenge and Xin (Twenge, 2000; S. F. Xin et al., 2020), the studies including cross-temporal meta-analysis must comply with specific inclusion criteria: (1) the study must use the GWB (General Well-Being) scale as the test questionnaire. (2) The subjects must all come from domestic tourists within China, meaning individuals who are residents of mainland China traveling within the country. (3) The study must have a minimum of 30 male and 30 female subjects. A small sample size and an imbalance in the male-to-female ratio can affect differences in well-being levels. (4) The study must include the mean (M), standard deviation (SD), and total sample size (N). For papers that present research data devoid of extensive study findings, the following are used for processing and the data are included in the database: Formula (1) ($\bar{x}=\frac{\sum x_{i}n_{i}}{\sum n_{i}}$, where $\bar{x}$ represents the combined mean, $x_{i}$ represents the mean, $n_{i}$ represents the sample size); and Formula (2) ($S_{T}=\sqrt{\frac{\left[\sum n_{i}S_{i}^{2}+\sum n_{i}{\left(x_{i}-\bar{x_{i}}\right)}^{2}\right]}{\sum n_{i}}}$, where $S_{T}$ represents the combined standard deviation, $S_{i}$ represents the standard deviation, ${\bar{x}}_{i}$ represents the combined mean, $x_{i}$ represents the mean, $n_{i}$ represents the sample size). (5) If identical data are released multiple times, the oldest instance is chosen. (6) Special stages or special groups should also be excluded as research subjects. The comprehensive information regarding the search strategy and selection procedure is illustrated in the PRISMA diagram presented in Figure 1.

As shown in Figure 1, this study identified 663 articles through the literature search, and 90 duplicate articles were removed during the initial screening. Subsequently, 198 articles were left after screening the titles and abstracts. Based on the specific inclusion criteria for this cross-temporal meta-analysis, 56 articles were ultimately selected for this study, including 17 journal articles and 39 theses and dissertations.

### 3.4. Control Variable Coding

The cross-temporal meta-analysis, akin to a conventional meta-analysis, is affected by the attributes of the literature. In addition to being influenced by publication years, it may also be influenced by factors such as the source of the literature (journal type) and the source of the participants (region) (Twenge, 2000; Twenge et al., 2016; Twenge & Campbell, 2001). The research results can be affected by these factors.

A cross-temporal meta-analysis is essentially a secondary study. The data used in this analysis are derived from original research. Based on the study objectives of the cross-temporal meta-analysis, it is essential to define the types of studies to be included and to make adjustments according to the prevailing circumstances. The caliber of the analysis in cross-temporal meta-analysis is intrinsically linked to the quality of the literature incorporated. It can be used as a basis for revising inclusion and exclusion criteria, explaining relationships between research variables, and assigning weights when merging data (T. S. Zhang, 2015).

Different types of literature may have different criteria for data inclusion. Consequently, establishing publication quality guidelines is essential to investigate if the link between two variables is affected by the type of literature. Similarly, differences in socio-economic development conditions across different regions may lead to different criteria for data inclusion. Therefore, it is necessary to control for regional differences to assess whether they have an impact on the relationship between two variables.

Regional variations and publication quality requirements may result in discrepancies in the reported data. Variable coding of data according to type of publication and region of participants (Z. Xin & Zhang, 2009). For publication standards, Chinese publications were classified based on the latest complete catalog of core Chinese journals at Peking University (2021 edition), while foreign publications were classified according to JCR subject category rankings, and some master’s and doctoral dissertations were also included. Therefore, the codes are as follows: 1 = core journal, 2 = general journal, 3 = master’s and doctoral dissertations. For regional classification, in accordance with the most recent standard classification by the National Bureau of Statistics, 0 = no clear regional information, 1 = eastern region, 2 = central region, 3 = western region, 4 = covering multiple regions (including studies with participants from two or more regions).

Table 1 illustrates that research about the well-being of Chinese tourists commenced in 2011. Because literature from other years did not meet the inclusion rules, the study period was narrowed down from 2000–2022 to 2011–2022, including a sample size of 42,312. Statistical results are reported for different genders, with 20,247 males and 22,065 females. The specifics of all studies chosen for meta-analysis are presented in Table 1.

### 3.5. Sources of Social Indicator Data

A cross-temporal meta-analysis can examine the evolution of tourists’ well-being across time and investigate the correlation between social macro variables and tourists’ well-being, elucidating the influence of social development and changes on their well-being. This involves selecting social indicators closely related to tourists’ well-being and conducting correlation or time-lag analysis with the mean level of tourists’ well-being.

This study adheres to the selection criteria established by (Twenge, 2000) to identify the relevant social indicators: (1) all social indicators can be easily quantified as important continuous variables; (2) all social indicators can truly explain the overall trend of change. Therefore, based on previous research by (S. F. Xin et al., 2020), this study selected two indicators of economic conditions: residents’ per capita income and residents’ consumption level, and three indicators of social connectedness: urbanization rate, employed population and health (life expectancy). The study seeks to elucidate the influence of social macro variables on tourist well-being by analyzing the role of five social indicators in predicting it. The names and data of these social indicators are obtained from the “China Statistical Yearbook” and the “China Tourism Statistical Yearbook”.

## 4. Results

### 4.1. Overall Changes in Tourists’ Well-Being over Time

**Figure 2:**
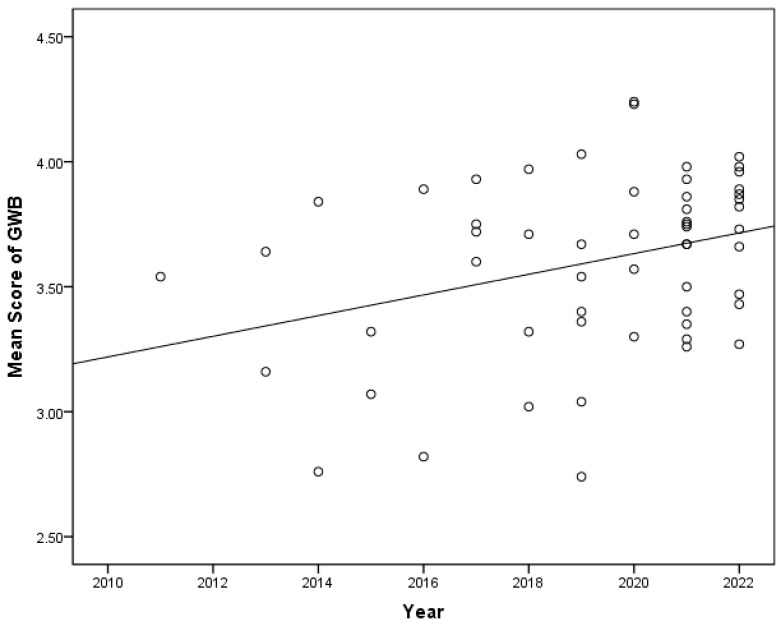
Changes in the mean (M) score of Chinese tourists’ well-being from 2010 to 2022.

The coded data were processed and analyzed using SPSS 21.0 software in this study. As illustrated in Figure 2, a scatter plot was generated using the years as the *x*-axis and the mean score of tourists’ well-being as the *y*-axis. This allowed for an intuitive description of the changes in tourists’ well-being over time. A rising trend was observed in the mean score of tourists’ well-being in China from 2011 to 2022. Furthermore, Pearson correlation analysis was performed to quantify the association between the mean score of tourists’ well-being and the number of years. The findings indicated a significant positive correlation between tourists’ well-being and time (*r* = 0.328, *p* < 0.05). Moreover, a regression equation and 95% *CI* were added to further verify the relationship between the two variables. The result revealed that tourists’ well-being showed an increasing trend with the years (*β* = 0.041, *R*^2^ = 11%, *p* < 0.05, 95% *CI* = (0.009, 0.074)). The *R^2^* value of 11% indicates that the year can explain 11% of the variation in tourists’ well-being. A *p*-value below 0.05 signifies that this regression equation is statistically significant at the 0.05 level. Furthermore, although the squared year variable yielded a significant result in the regression equation, the curve exhibited minimal steepness. Consequently, no additional analyses for curvilinear effects were performed.

In addition to the influence of the year on the changes in tourists’ well-being, cross-temporal meta-analysis is also affected by factors such as publication type and survey area (Z. Xin & Zhang, 2009). In order to control for these variables, this study conducted a multiple linear regression analysis using SPSS 21.0 software to explain the characteristics of tourists’ well-being changes over time. Initially, tourists’ well-being served as the dependent variable, while publication type and survey area were designated as independent variables in the first-level regression analysis, referred to as Model 1. At the same time, the year was included as an independent variable in the second-level regression analysis, which was set as Model 2. As shown in Table 2, both Model 1 (*F*1 = 4.407, *p* < 0.05) and Model 2 (*F*2 = 5.807, *p* < 0.01) were statistically significant. The *R*^2^ value was 14.3% in Model 1 and 25.1% in Model 2, indicating that the appearance of the year increased the explanatory power of the model by 10.8%, and the influence of the year on tourists’ well-being was greater. When controlling for publication type and survey area, the results showed (*β* = 0.36, *p* < 0.01, 95% *CI* = (0.012, 0.079)), further demonstrating that the relationship between tourists’ well-being and year in China was not affected by variables such as publication type and survey area. In summary, the well-being of Chinese tourists showed a significant upward trend from 2011 to 2022.

### 4.2. Magnitude of Changes in Tourists’ Well-Being over Time

As stated in the above conclusion, the level of well-being among Chinese tourists has been on the rise over the years. However, it is not clear how much the level of well-being has increased between 2011 and 2022. Consequently, a regression equation adjusted for sample size is employed to compute the mean scores of tourist well-being for the first year (2011) and the final year (2022) encompassed in the study. The specific operation is as follows: First, the regression equation is used to calculate the mean score of tourist well-being for a specific year (*y* = *bx* + *c*, where *b* is the partial regression coefficient, *c* is the intercept or constant, *x* is the year, and *y* is the average total mean score of tourist well-being). The regression equation can obtain the average total mean score of tourist well-being for 2011 and 2022. Second, use Formula (3) ($d=\frac{M_{2022}-M_{2011}}{M_{SD}}$, where d refers to effect size, $M_{2022}$ refers to the average total mean score of tourist well-being in 2022, $M_{2011}$ refers to the average total mean score of tourist well-being in 2011, $M_{SD}$ refers to the average standard deviation value between 2011 and 2022) to obtain the effect size of the change in the mean of tourist well-being. After calculation, the mean of tourist well-being in 2011 and 2022 in China was 2.57 and 3.02, respectively. The mean score of tourist well-being increased by 0.45 points during this period, and the average standard deviation was 0.85 (*M_SD_* = 0.85). The effect size *d* was 0.53 (*d* = 0.53), indicating an increase of 0.53 standard deviations. According to (Cohen, 1992), when *d* = 0.80 (absolute value), the effect size is large; when *d* = 0.50 (absolute value), the effect size is medium; and when *d* = 0.20 (absolute value), the effect size is small. Based on the results obtained in this article, the increase in the mean of tourist well-being in China is between the medium and large effect sizes. At the same time, when using Formula (4) ($r^{2}={\left(\frac{d}{\sqrt{d^{2}+4}}\right)}^{2}$, where d refers to effect size) to calculate the variance explained by the effect size *d* as a function of years, the result *r*^2^ is 6.6%. In other words, the year can account for 6.6% of the variance in tourist well-being.

### 4.3. Correlations Between Mean Scores of Chinese Tourists’ Well-Being and Social Indicators

The results of this study indicate a significant upward trend in the level of well-being among Chinese tourists from 2011 to 2022. What are the factors that contribute to this upward trend? As mentioned in the previous literature, the correlation between the level of tourist well-being and social indicators can provide a possible explanation for this upward trend. To better understand how social change and development impact psychological variables, the cross-temporal meta-analysis method can shed light on the trends in psychological variables’ development and the connections between individual psychological variables and social indicators. Time-lag analysis was used to demonstrate that the mean scores of social indicators (such as residents’ per capita income, residents’ consumption level, etc.) are closely related to the psychological indicators of the target population (such as tourists). If the coefficients between the mean score of tourist well-being and the scores of social indicators (such as residents’ per capita income, residents’ consumption level, urbanization rate, employed population and life expectancy) from the current year and the past 3 or 5 years are significant, it is considered that social indicators can predict changes in the level of tourist well-being. A time lag of 3 to 5 years substantiates the validity of the predictive influence of societal change on tourist well-being levels.

As shown in Table 3, it is reasonable that the time lag of 5 or 3 years helps us confirm the predictive effect of social change on the SWB level (S. F. Xin et al., 2020). The correlation between the mean score of the well-being level of Chinese tourists and the scores of five social indicators is almost significant at the current year, 3 years ago, and 5 years ago, showing a positive correlation. In other words, economic conditions (residents’ per capita income and residents’ consumption level) and social connectedness (urbanization rate, employed population and life expectancy) are all important factors that affect the rising well-being level of Chinese tourists.

## 5. Discussion and Conclusions

### 5.1. The Level of Chinese Tourists’ Well-Being Is Showing an Upward Trend

This study performed a cross-temporal meta-analysis of 56 papers utilizing the GWB scale to examine the evolution and fluctuations in well-being levels among Chinese tourists from 2011 to 2022. Firstly, the preliminary linear regression revealed a substantial positive association between the mean level of well-being of Chinese tourists and the year, signifying a general upward trend in their well-being over the preceding decade. Next, using a weighted regression equation, the study calculated the rate of change and found an increase of 0.53 standard deviations. This further validates the impact of China’s rapid economic development and macro-social factors (urban development, social security, culture, education, etc.) on the overall rise in well-being levels among Chinese residents (Xing & Hu, 2022). Self-Determination Theory (SDT) suggests that the level of satisfaction with core psychological needs (autonomy, competence, and relatedness) has an impact on well-being (Ryan & Deci, 2000). Economic growth and urbanization in China have expanded opportunities for individuals to make self-directed choices in their lives. Increased disposable income and access to diverse leisure activities (e.g., travel) have allowed Chinese tourists to engage in activities that align with their personal interests and values. China’s economic and social advancements have also fostered opportunities for individuals to develop and demonstrate competence. Exposure to new experiences through travel and cultural exchange has provided opportunities for learning and mastery, further boosting feelings of competence. Social and cultural developments in China have strengthened interpersonal connections and community ties, which are essential for well-being. The upward trend in well-being among Chinese tourists reflects not only economic progress but also the fulfillment of fundamental psychological needs, which are essential for sustained well-being.

### 5.2. Impact of Social Changes on Tourists’ Well-Being

This study used a cross-temporal meta-analysis to elucidate the macro-social elements affecting the enhancement of tourists’ well-being by examining its underlying causes horizontally. The research results show that the economic conditions (residents’ per capita income and residents’ consumption level) and social connectedness (urbanization rate, employed population and life expectancy) are closely related to the upward trend of Chinese tourists’ well-being level.

### 5.3. The Rise in Well-Being Levels Among Chinese Tourists May Be Related to Changes in the Economic Conditions

This study discovered that two indicators of the economic conditions (residents’ per capita income and residents’ consumption level) have a substantial beneficial influence on the well-being levels of tourists. The two economic conditions (residents’ per capita income and residents’ consumption level) from the current year, three years ago, and five years ago were all found to have a strong positive predictive influence on tourist well-being. Thus, an increase in income and consumption enhances the feeling of well-being (Jaikumar et al., 2018).

The per capita income of residents significantly enhances the well-being of tourists. Following the reform and opening up, China has had decades of rapid economic expansion, with its GDP attaining 17.9 trillion dollars in 2022, representing 14.4% of the global total and establishing itself as the world’s second-largest economy. The swift economic growth enhances per capita income for locals, hence boosting tourism demand and creating additional opportunities for prospective travelers (Liu et al., 2013). Increased travel opportunities and frequency of travel enhance people’s subjective well-being (Zheng et al., 2022). Therefore, an increase in residents’ per capita income can effectively enhance tourists’ well-being (Diener et al., 1993, 2018).

The consumption levels of residents positively influence the well-being of tourists. The correlation between well-being and consumption has consistently been a prominent subject of investigation. The rapid economic development will gradually change people’s lifestyles, and their consumption will shift from pursuing quantity to pursuing quality. The consumption structure will gradually optimize from a low-level structure to a high-level structure (Ning, 2000). As a middle- to high-level consumption activity, tourism reflects people’s desire for a better life. Relevant studies indicate that as the level of consumption increases, household consumption transitions from survival-oriented to enjoyment-oriented, and high-income individuals derive greater well-being from enjoyment-oriented consumption (Rao et al., 2019). As a kind of enjoyment consumption project, people can obtain a deeply spiritual experience, find fun and happiness, and achieve a sense of well-being through tourism (H. Zhang, 2022). Data from the Ctrip Travel website show that Chinese people, especially those in the middle- and high-income groups, believe that consumption brings the strongest well-being in tourism. Consequently, an elevated degree of consumption correlates with enhanced well-being among tourists (Noll & Weick, 2015).

### 5.4. The Rise in Well-Being Levels Among Chinese Tourists May Be Closely Related to Social Connectedness

The research results indicate that three social indicators associated with social connectedness (urbanization rate, employed population, and life expectancy) are highly correlated with the increase in well-being among Chinese tourists. Furthermore, these three social connectedness indicators (urbanization rate, employed population, and life expectancy) positively predict tourist well-being in the current year as well as three and five years prior.

The degree of urbanization positively influences the well-being of tourists. Following the reform and opening up, China’s urbanization has progressed swiftly, achieving an urbanization rate of 64.72% by 2022 (K. Wang et al., 2016). The increase in urbanization level has promoted the upgrading of tourism consumption demand and the expansion of investment in tourism public service facilities and infrastructure construction, providing a high-quality consumption environment and a convenient, comfortable tourism environment for tourists (Lin & Zhao, 2020). The advancement of urbanization concurrently promotes the tourism consumption capacity and enhances the quality of public tourism services, consequently improving tourists’ well-being (Shen et al., 2023). Consequently, the swift advancement of urbanization significantly influences the enhancement of tourists’ well-being (Navarro et al., 2020).

The overall current employment situation has a positive effect on the well-being of tourists. The growth in the employed population not only leads to higher personal income but also provides opportunities for individuals to fulfill their travel desires through their work engagements. Employed individuals can fulfil their physical and mental health requirements through health tourism, including medical treatment, rehabilitation, exercise, and recovery, while participating in business and official meetings (Guo & Ni, 2015). The relationship between occupation and economic income is closely related, and the amount of money that people in different occupations can freely dispose of varies significantly. Increased income correlates with elevated tourism motivation (F. Qiu, 1996). In addition, the increase in the employed population allows people to have income from their labor. Longer working hours also force people to seek novelty and well-being by escaping from the usual environmental pressures and complex interpersonal relationships. Therefore, tourism has become a way for many professional groups to pursue well-being.

Tourists’ well-being improves as their life expectancy increases. Since the inception of the People’s Republic of China, the average life expectancy has risen from 35 years to 78 years in 2022, effectively doubling over the years. This improvement in average life expectancy provides the basic physical conditions for people to travel and enjoy a better life. Poor health conditions are often barriers to participating in sports activities and tourism. Maintaining health and physical fitness should be emphasized to enhance a positive tourism experience (Rowinski et al., 2017). Nutritious dietary practices, physical activity, and psychological elements profoundly affect the health and well-being of elderly individuals (Guan et al., 2017). Therefore, as the average life expectancy in China continues to increase and people’s health awareness grows stronger, the opportunities for outbound tourism increase, leading to a continuous improvement in the well-being of Chinese tourists (Evans & Soliman, 2019; Pourabdol et al., 2015).

Thus, it is important to consider the aforementioned research and discussions when trying to predict the psychological variables of specific groups, like the tourists’ well-being in this study, because these macro-factors are indicative of social development status and are constantly changing. These factors include things like residents’ per capita income, residents’ consumption levels, urbanization rates, employed population and life expectancy. The findings from cross-temporal meta-analysis can concentrate on the macro-social indicators associated with psychological variables and offer precise insights for policies tailored to specific psychological factors, such as well-being, which is crucial for policymakers. At the same time, this study combines both longitudinal (time dimension) and cross-sectional (macro-social indicators) perspectives, which makes the research method more comprehensive and enriches the theory of tourists’ well-being.

## 6. Research Contribution

### 6.1. Theoretical Contribution

The first contribution is to clarify the trend of the well-being level among Chinese tourists at the group level. This study examines the trend of the psychological variable of well-being from the viewpoint of tourists. Given the high mobility of tourists and their dynamic behavior, it is difficult to longitudinally track changes in a psychological variable for tourists (Kim et al., 2019b). This study uses secondary data from the literature to explore the trend of well-being among tourists, thereby overcoming the difficulty of tracking the changes in tourist well-being. This study gathered material pertaining to travelers’ well-being from 2011 to 2022, except for 2012 due to the absence of pertinent literature, and produced relevant indicators reflecting annual variations in tourists’ well-being. Secondly, each piece of research literature studies “tourists” as a unit. Due to previous research on tourists in different backgrounds and case studies (H. Q. Qiu et al., 2022; Reitsamer & Brunner-Sperdin, 2017; L. J. Su et al., 2022), it is challenging to represent the changing characteristics of the “group” of tourists. The greatest innovation of this research is using a cross-temporal meta-analysis method, taking each piece of previous research as a research object and the “group” of tourists each year as a change scale. The 56 research studies conducted across China over 11 years enhance the representation of the well-being status of the tourist “group”, hence expanding the comprehension of group psychological characteristics horizontally.

The second contribution is to ascertain the primary elements influencing the well-being of Chinese tourists. In the field of social psychology, many researchers point out that social psychology or social mentality (including social emotions) is a group phenomenon that is not equivalent to the accumulation of individual psychology (Lan & Pu, 2016). Therefore, when discussing various factors that influence tourist well-being, we should not ignore the abstract concept of the group, that is, the characteristics, properties, and values of the tourist group. More importantly, the social psychology of a group is not a static concept of traits. It should be viewed from a constructionist perspective, with the group constantly processing information in the surrounding environment, while the external social environment is changing with the times. This study examined five elements that may influence tourist well-being, based on China’s social development features over the past decade, and employed a time-lag analysis method to ascertain any association among them. This study proves that the five indicators of residents’ per capita income, residents’ consumption level, urbanization rate, employed population and life expectancy, are important social factors affecting the well-being of Chinese tourists and continue to affect the change in tourist well-being over time.

### 6.2. Practical Contribution

Our research additionally offers the subsequent practical implications. First, it provides policy guidelines for tourism destination managers. Our findings confirm the positive impact of residents’ per capita income, residents’ consumption level, urbanization rate, employed population and life expectancy on the well-being of tourists. Policymakers can implement measures such as enhancing social security systems, instituting paid yearly leave for employees, expediting the expansion of high-quality medical services, and upgrading basic living facilities for inhabitants to promote travel. This action is anticipated to enhance the well-being and social welfare of the Chinese populace. Secondly, our research demonstrates that the current tourist products, services, and facilities in China do not meet the needs of all market segments. Tourist firms ought to build distinct tourist projects aimed at certain demographics throughout the creation of tourism products, thus augmenting the personalization and distinctiveness of these offerings. Group-based experiences (e.g., family or community vacations) may have a greater impact on well-being in collectivist cultures than in individualistic societies, where individual achievement and autonomy may have a greater impact on well-being. We will adapt tourism policies and product development in other countries to cultural differences. For example, in countries with high levels of cultural heritage tourism, such as Greece or Italy, experiences that emphasize the promotion of personal connections to history or local culture may be more effective in increasing well-being than destinations that focus on modern, high-tech infrastructure.

## 7. Limitations and Future Research

Despite the valuable results attained in this investigation, certain limitations persist. Firstly, in addition to the GWB scale, alternative measures for assessing well-being include the Happiness Index Scale (HIS), the Psychological Well-being Scale (PWS), and the Multiple Happiness Questionnaire (MHQ). Cross-temporal meta-analysis may be test other scales to do comparative research with the GWB scale. Secondly, this study examined the temporal trends in the well-being levels of Chinese tourists by picking pertinent social indicators for macro analysis. The causal association between each social macro-factor and tourist well-being has not been validated using longitudinal approaches, nor has the precise influencing mechanism been thoroughly investigated. While there may be correlations between well-being and social indicators, this does not imply a direct causal link. The findings should be interpreted with caution, as there may be other factors at play, such as reverse causality, where changes in well-being could influence social indicators, or unobserved confounders that drive both variables simultaneously. Therefore, future research can study the internal relationships of macro-factors affecting the well-being of Chinese tourists to reveal the underlying mechanisms of their influencing factors. Furthermore, this study has confirmed the relationship between rapid economic development, social macro-factors, and tourists’ well-being in China. Further exploration can be made in the future based on the hypothesized relationship between these internal factors and tourists’ well-being.

## Figures and Tables

**Table 1:** Fundamental details of the source literature and variable coding assignment table.

NO.	Authors	Publication Year	Sample Size	Number of Male Groups	Number of Female Groups	Publication Class	Region	GWB (M)	GWB (SD)
1	Gao	2011	366	174	192	3	2	3.54	0.69
2	Zhao	2013	95	59	36	3	3	3.16	0.48
3	Gong	2013	190	32	158	3	2	3.64	0.81
4	Zhang	2014	491	228	262	2	3	3.84	NA
5	Ruan	2014	381	185	196	3	4	2.76	1.20
6	Wang	2015	317	103	206	3	1	3.32	1.37
7	Yan	2015	385	202	183	3	1	3.07	NA
8	Xiang	2016	373	186	187	3	1	2.82	1.27
9	Su et al.	2016	451	227	224	1	2	3.89	0.69
10	Ma & Zhang	2017	280	118	162	1	4	3.75	0.74
11	Yang	2017	227	124	103	3	1	3.6	0.72
12	Zhan	2017	261	110	151	3	2	3.93	0.91
13	Lv & Xie	2017	576	300	276	1	4	3.72	1.04
14	Zhang	2018	174	77	97	3	3	3.97	0.47
15	Wang	2018	617	297	320	3	1	3.71	1.15
16	Zheng	2018	259	110	149	3	1	3.02	0.55
17	Zhang et al.	2018	165	70	96	2	1	3.32	NA
18	Chen & Li	2019	306	152	154	2	1	3.54	0.71
19	Zhang	2019	703	296	407	3	2	4.03	0.89
20	Fu	2019	225	119	106	3	3	3.04	1.37
21	Zhou	2019	256	108	148	3	1	3.36	0.98
22	Meng	2019	610	269	341	3	2	3.67	0.99
23	Gao	2019	366	174	192	3	3	3.4	0.07
24	Tang	2019	294	110	184	3	4	2.74	1.16
25	Wang et al.	2020	332	181	151	1	1	4.23	0.73
26	Liu	2020	207	89	118	3	1	3.57	NA
27	Lian	2020	753	326	427	3	4	3.3	1.47
28	Liu	2020	401	164	237	3	4	3.71	0.64
29	Kim et al.	2020	742	330	412	1	0	4.24	0.92
30	He et al.	2020	541	259	282	1	2	3.88	0.7
31	Wang et al.	2021	345	162	183	2	1	3.40	NA
32	Feng	2021	302	135	167	3	1	3.76	NA
33	Shao	2021	388	181	207	3	1	3.74	0.86
34	Ma	2021	364	157	207	3	1	3.93	0.82
35	Jiang	2021	421	204	217	3	2	3.26	0.41
36	Lv	2021	474	207	267	3	2	3.81	1.12
37	Shao	2021	11,726	6166	5560	3	4	3.86	0.85
38	Cao	2021	542	188	354	3	4	3.35	1.03
39	Zhang	2021	261	138	123	3	1	3.98	0.6
40	Tu	2021	310	117	193	3	2	3.29	1.03
41	Bai	2021	316	200	116	3	3	3.67	NA
42	Sun	2021	400	173	227	3	3	3.67	0.94
43	Li et al.	2021	414	218	196	1	3	3.75	0.92
44	Cheng & Xu	2021	399	231	168	1	3	3.5	0.75
45	Feng & Yang	2022	271	122	149	2	1	3.73	0.81
46	Zhou et al.	2022	318	123	195	1	1	3.96	0.53
47	Guan & Cheng	2022	260	100	160	1	1	3.85	0.74
48	Guan	2022	460	191	269	3	1	3.27	NA
49	Cao	2022	220	108	112	3	2	3.82	0.59
50	Leng	2022	226	108	118	3	1	3.47	NA
51	Qiao	2022	385	209	176	3	2	3.89	1.07
52	Kuang	2022	306	132	174	3	1	3.98	1.04
53	Wu	2022	212	108	104	3	3	3.43	1.06
54	Chen	2022	384	130	254	3	3	3.66	0.78
55	Zheng et al.	2022	10,953	5126	5827	1	4	3.87	0.82
56	Peng et al.	2022	311	134	177	1	1	4.02	0.57

Note: Region: 0 = no definite region information; 1 = east; 2 = center; 3 = west; 4 = multiple. Publication class: 1 = core journal; 2 = publication from other academic sources; 3 = dissertations and master’s theses. NA, missing values; M, mean score of tourist well-being; SD, standard deviation of tourist well-being.

**Table 2:** Multiple regression analysis of predictor variables for General Well-Being (GWB).

Variable	Dependent Variable: GWB Score							
	Model One				Model Two			
	*F*1	*R* ^2^	95% *CI*	*β*1	*F*2	*R* ^2^	95% *CI*	*β*2
Publication class	4.407 **	0.143 **	(−0.167, −0.022)	−0.335 **	5.807 ***	0.251 ***	(−0.142, −0.001)	−0.253 **
Region			(−0.026, 0.084)	0.135			(−0.05, 0.06)	0.024
Publication year							(0.012, 0.079)	0.36 ***

** *p* < 0.05, *** *p* < 0.01.

**Table 3:** Correlations between the mean scores of Chinese tourist well-being and social indices.

	Actual Year		Three Years Prior		Five Years Prior	
Social Indicators	*β*	95% *CI*	*β*	95% *CI*	*β*	95% *CI*
Residents’ per capita income	0.398 ***	(2.302, 3.484)	0.406 ***	(2.455, 3.495)	0.311 **	(2.725, 3.691)
Residents’ consumption level	0.386 ***	(2.365, 3.517)	0.407 ***	(2.535, 3.507)	0.41 ***	(2.6, 3.516)
Urbanization rate	0.395 ***	(0.012, 0.076)	0.389 ***	(0.009, 0.062)	0.409 ***	(0.01, 0.058)
Employed population	−0.414 ***	(8.774, 30.809)	−0.376 **	(11.819, 65.691)	0.163	(−69.459, 26.103)
Life expectancy	0.409 ***	(0.052, 0.286)	0.378 **	(0.046, 0.34)	0.402 ***	(0.057, 0.335)

** *p* < 0.05, *** *p* < 0.01.

## Data Availability

The data presented in this study are available upon request from the corresponding author.
